# Markers of abnormal tissue deformation and fibrosis in remote myocardium following acute myocardial infarction: a comparison of diabetics versus non-diabetics performed using spatially matched 4D strain and native T1 mapping

**DOI:** 10.1186/1532-429X-18-S1-O6

**Published:** 2016-01-27

**Authors:** Alessandro Satriano, Kate Fenwick, Dexter D Waters, Haris Vaid, Yoko Mikami, Naeem Merchant, Carmen P Lydell, Andrew G Howarth, Teresa A Whitman, Derek V Exner, Bobak Heydari, Nowell M Fine, James A White

**Affiliations:** 1 Stephenson Cardiac Imaging Centre, Calgary, AB Canada; 2 grid.22072.350000000419367697Division of Cardiology, School of Medicine, University of Calgary, Calgary, AB Canada; 3 grid.410356.50000000419368331Queen's University, Kingston, ON Canada; 4 grid.14709.3b0000000419368649McGill University, Montréal, QC Canada; 5 grid.22072.350000000419367697University of Calgary, Calgary, AB Canada; 6 grid.22072.350000000419367697Department of Diagnostic Imaging, University of Calgary, Calgary, AB Canada; 7 grid.419673.eMedtronic, Inc., Minneapolis, MN USA; 8 Libin Cardiovascular Institute of Alberta, Calgary, AB Canada

## Background

Late Gadolinium Enhancement (LGE) MRI accurately delineates regions of irreversible injury following myocardial infarction (MI). However, markers of tissue abnormalities in the remote, non-infarcted myocardium remain poorly investigated. In this study we explore the utility of 4D strain analysis and native T1 mapping to identify alterations in tissue function or characteristics in diabetics following MI.

## Methods

91 consecutive patients ≥3 months post MI were enrolled. All patients underwent an imaging protocol at 1.5T using standard multi-planar cine imaging, pre-contrast T1 mapping (shMOLLI) and LGE imaging. An additional 40 normal controls were studied. 4D Strain analysis using a deformation field approach was performed using in-house software (GIUSEPPE, developed in Matlab R2015a) to obtain segmental estimates of Peak and Time to Peak strain of the left ventricle (LV). Strains were calculated in Radial (Err), Circumferential (Ecc), Longitudinal (Ell) and Principal directions (Emin and Emax). Strain systolic amplitude and time to peak (ttp) indexed by RR duration were also obtained. Spatially matched segmental estimates of %LGE and native T1 (ms) were obtained using cvi42 (Circle Cardiovascular, Calgary). Segmental strain values of the study population were indexed to reference segmental values obtained from normal controls. All segments with <5 LGE% were considered eligible as non-infarcted or "remote" myocardium and their indexed strain values then compared between study patients with (N = 29) and without (N = 62) type 2 diabetes. Finally, native T1 of remote tissue was compared for the same cohorts.

## Results

Mean age was 60.6+/-9.6 with mean LV EF of 42.1+/-7.1%. Mean Segmental values for remote myocardial strain were significantly reduced in the study cohort versus normal controls using Peak Longitudinal (Ell), Minimum Principal (Emin) and Max Principal (Emax) Strain (p < 0.05). Diabetics showed a significant (p < 0.05) reduction in remote tissue strains relative to non-diabetics (Ell: -19.00+/-16.15 vs. 8.96+/-15.46% reduction, Emin: 16.01+/-14.56 vs. 5.62+/-14.17% reduction, Emax: 57.05+/-12.70 vs. 45.69+/-22.33% reduction), as well as significant increase in time to peak systolic strain (ttpErr: 11.40+/-18.64 vs. 3.39+/-20.17%, ttpEcc: 3.91+/-18.18 vs. -3.19+/-12.62%, ttpEmin: 9.34+/-14.55 vs. -2+/-12.77%). A trend towards longer native T1 values, suggestive of interstitial fibrosis, was found among remote myocardium of diabetics versus non-diabetics (1000+/-44.01 vs. 991+/-38.55 ms, p = 0.28).

## Conclusions

4D strain analysis demonstrates impaired myocardial performance, consistent with underlying tissue remodelling, in remote myocardial segments in diabetics following acute myocardial infarction. This finding was associated with a trend towards prolonged native T1 values, a surrogate marker of interstitial fibrosis. Study into the prognostic utility of this marker for future adverse cardiovascular events is required.

